# Competition Among *Gardnerella* Subgroups From the Human Vaginal Microbiome

**DOI:** 10.3389/fcimb.2019.00374

**Published:** 2019-10-31

**Authors:** Salahuddin Khan, Maarten J. Voordouw, Janet E. Hill

**Affiliations:** Department of Veterinary Microbiology, Western College of Veterinary Medicine, University of Saskatchewan, Saskatoon, SK, Canada

**Keywords:** *Gardnerella*, vaginal microbiome, interaction, bacterial vaginosis (BV), competition, microbial ecology, biofilm

## Abstract

*Gardnerella* spp. are hallmarks of bacterial vaginosis, a clinically significant dysbiosis of the vaginal microbiome. *Gardnerella* has four subgroups (A, B, C, and D) based on cpn60 sequences. Multiple subgroups are often detected in individual women, and interactions between these subgroups are expected to influence their population dynamics and associated clinical signs and symptoms of bacterial vaginosis. In the present study, contact-independent and contact-dependent interactions between the four *Gardnerella* subgroups were investigated *in vitro*. The cell free supernatants of mono- and co-cultures had no effect on growth rates of the *Gardnerella* subgroups suggesting that there are no contact-independent interactions (and no contest competition). For contact-dependent interactions, mixed communities of 2, 3, or 4 subgroups were created and the initial (0 h) and final population sizes (48 h) were quantified using subgroup-specific PCR. Compared to the null hypothesis of neutral interactions, most (69.3%) of the mixed communities exhibited competition. Competition reduced the growth rates of subgroups A, B, and C. In contrast, the growth rate of subgroup D increased in the presence of the other subgroups. All subgroups were able to form biofilm alone and in mixed communities. Our study suggests that there is scramble competition among *Gardnerella* subgroups, which likely contributes to the observed distributions of *Gardnerella* spp. in vaginal microbiomes and the formation of the multispecies biofilms characteristic of bacterial vaginosis.

## Introduction

*Gardnerella vaginalis* is considered a hallmark of bacterial vaginosis, a dysbiosis of the vaginal microbiome, although it is also commonly detected in women who do not meet the clinical criteria for vaginosis. *Gardnerella* comprises four sub-groups (A, B, C, and D), based on cpn60 barcode sequences and whole-genome sequences (Paramel Jayaprakash et al., 2012; Schellenberg et al., 2016). These subgroups have also been classified as clades 1–4 (Ahmed et al., 2012), with subgroups A, B, C, and D corresponding to clades 4, 2, 1, and 3, respectively. More recently, Vaneechoutte et al. amended the description of *Gardnerella* and defined three new species within the genus: *G. leopoldii, G. swidsinskii*, and *G. piotti* (Vaneechoutte et al., 2019). These species correspond to three of the previously defined subgroups: *G. vaginalis* (subgroup C/clade 1), *G. leopoldii* and *G. swidsinskii* (subgroup A/clade 4), and *G. piotii* (subgroup B/clade 2), while subgroup D/clade 3 encompasses several unnamed “genome species.”

Phenotypic differences between the subgroups have been identified that could influence the role of *Gardnerella* spp. in the vaginal microbiome and their contributions to establishment and maintenance of vaginal dysbiosis (Schellenberg et al., 2016; Janulaitiene et al., 2018; Vaneechoutte et al., 2019). Furthermore, it has been shown that clades 4, 1, and 3 (Subgroups A, C, and D) are more often associated with bacterial vaginosis as defined by a high Nugent score or Amsel's criteria (Albert et al., 2015; Hilbert et al., 2017). Subgroup B or Clade 2 has been reported to be more abundant in women with an intermediate Nugent score (Balashov et al., 2014; Albert et al., 2015; Hilbert et al., 2017). Taken together, these observations highlight the potential clinical significance of the composition of the *Gardnerella* community within the vaginal microbiome.

*Gardnerella* clades or subgroups can be reliably distinguished in vaginal microbiome profiles using cpn60 barcode sequences (Hill et al., 2005; Paramel Jayaprakash et al., 2012), whereas 16S rRNA gene sequencing does not provide sufficient resolution (Vaneechoutte et al., 2019). Profiling of vaginal microbiomes using cpn60 barcode sequencing, and application of clade-specific PCR has shown that the vagina is often colonized by multiple subgroups simultaneously (Albert et al., 2015; Hilbert et al., 2017). The relative abundances of these subgroups, however, are not equal, and one subgroup usually dominates. The combinations and relative abundances of cpn60-defined subgroups of *Gardnerella* have been used to define previously undescribed population structures called community state types (CST) in the human vaginal microbiome (Albert et al., 2015). Given the observed phenotypic diversity within *Gardnerella*, an understanding of the factors that determine *Gardnerella* population structure in the vaginal microbiome is critical.

Potential factors contributing to the relative abundance patterns of *Gardnerella* subgroups in the vaginal microbiome include differences among subgroups in terms of biofilm formation, adhesion, overall fitness, and resistance to anti-bacterial factors (either produced by other microbiota or delivered as a medical intervention). Interactions between the subgroups may influence the population dynamics of the *Gardnerella* subgroups (Czárán et al., 2002; Hibbing et al., 2010; Faust and Raes, 2012). When the vaginal microbiome is dominated by *Gardnerella*, interactions between subgroups would be more frequent than with other bacterial species because they are closely related and therefore more likely to occupy the same niche (Darwin, 1859). *Gardnerella* can form biofilm in isolation and can also be incorporated in multispecies biofilms in the vagina (Hardy et al., 2015, 2016). Inter- and intraspecies interactions are ubiquitous within such multispecies biofilms (Narisawa et al., 2008; Elias and Banin, 2012; Burmølle et al., 2014), and such interactions may lead to competitive exclusion (Kerr et al., 2002; Oliveira et al., 2015). Thus, it is possible that competition between *Gardnerella* subgroups within the biofilm shapes the microbial population structure in the vaginal microbiome.

Competition between subgroups could take the form of a contest where two subgroups interact directly in either a contact-dependent manner or a contact-independent manner involving the secretion of effectors that reduce the fitness of competitors (Stubbendieck and Straight, 2016). Direct interactions can either inhibit the growth of one or more competitor(s) (Hayes et al., 2014; Willett et al., 2015), or trigger an enhanced biofilm response (Oliveira et al., 2015; Ren et al., 2015). In either case, competition could result in the exclusion of one or more competitor(s). Alternatively, competition between closely related taxa may take the form of a scramble (Hibbing et al., 2010), where they do not interact directly, but one competitor outgrows the others through its superior ability to use shared resources, such as nutrients. In a scramble mode of competition, all competitors have to share finite resources, which can reduce the fitness of competing organisms. This type of competition is often referred to as non-interfering exploitative competition (Russel et al., 2017).

The objective of our study was to seek evidence of contact-independent or contact-dependent interactions between *Gardnerella* subgroups that affect growth *in vitro*. Our results demonstrate that strains representing *Gardnerella* subgroups A, B, C, and D can coexist in biofilms but that mixing of subgroups does not enhance biofilm formation. Our findings also suggest the presence of a non-interfering, exploitative competition in mixed subgroup communities of *Gardnerella*.

## Methods

### *Gardnerella* Isolates

Isolates of *Gardnerella* spp. used in this study were drawn from a previously described culture collection kept at −80°C (Schellenberg et al., 2016) (Table S1). The subgroup affiliations of all isolates were determined by cpn60 barcode sequencing (Links et al., 2012). Selected isolates were revived from −80°C on Columbia agar plates with 5% sheep blood and incubated under anaerobic conditions (BD GasPak EZ Anaerobe Gas Generating Pouch System, NJ, USA) at 37°C for 48 h.

### Measurement of the Effect of *Gardnerella* Culture Supernatant on Growth

The purpose of this part of the study was to test whether the *Gardnerella* subgroups produce molecules that inhibit the growth of the other subgroups (i.e., contact-independent interactions). Specifically, we wanted to test the effect of cell-free supernatant from *Gardnerella* subgroups on the growth and biofilm formation of the other subgroups. To detect contact-independent interactions, we tested a total of 56 combinations using 14 isolates of subgroups A (*n* = 4), B (*n* = 4), C (*n* = 3), and D (*n* = 3) (Table 1). Isolates that were used to derive cell-free supernatant (CFS) were called producer strains, and strains on which the effect of prepared CFS was tested were called focal strains. Selected focal strains of all four subgroups were grown in medium containing 10% CFS from producers belonging to other subgroups and their own subgroup (“self-CFS”). Experiments were performed in two culture media: NYC III broth, which is recommended by the American Type Culture Collection (ATCC) for *Gardnerella* culture, and BHI + 1% glucose broth, to determine if culture media influenced growth of *Gardnerella* subgroups in the presence or absence of CFS.

**Table 1:** Numbers of combinations of CFS producers and focal subgroups tested to detect contact-independent interactions.

**Focal subgroup**	**CFS producer subgroup**			
	**A**	**B**	**C**	**D**
A	3	3	3	3
B	4	4	4	4
C	4	4	4	4
D	3	3	3	3

To produce the CFS, colonies from Columbia blood agar plates were harvested using sterile swabs, resuspended in 5 ml of NYC III broth and incubated anaerobically for 72 h at 37°C to reach stationary phase. CFS was generated by centrifuging the broth culture at 3,000 × g for 30 min (Jung et al., 2014). The supernatant was filter-sterilized using 0.22 μm filters and was used on the same day. Filter-sterilized CFS was streaked on Columbia blood agar plates to confirm sterility.

To test the effect of CFS on the focal strains, colonies from blood agar plates were harvested using sterile swabs, resuspended in 0.85% saline and adjusted to McFarland turbidity standard 1. Fifteen microliters of each test strain suspension were added to 135 μl of NYC III or BHI + 1% (v/v) glucose and 10% (v/v) CFS in individual wells of a flat bottom 96-well plate (Corning Costar, NY, USA). The focal strains were also grown in control wells with media containing no CFS. Negative controls consisted of 15 μl of 0.85% saline added to 135 μl of culture media (NYC III or BHI + 1% glucose) and sterile culture media alone. To confirm the viability of the inocula, focal strain suspensions were streaked on to Columbia blood agar. Each combination of CFS and the focal strain was performed in three technical replicates.

### Quantification of Total Growth, Planktonic Growth, and Biofilm Growth

Total growth, planktonic growth, and biofilm formation were determined for each combination at 48 h (Figures S1–S14). Total growth was calculated as the difference in optical density measured at 595 nm between the 48 h and the 0 h time points. The OD_595_ was measured using a VMax Kinetic ELISA Absorbance Microplate Reader (Molecular Devices, CA, USA). Planktonic growth was measured by transferring 150 μl of supernatant from each well to a fresh 96-well plate and determining the OD_595_. To measure the biofilm formation, a crystal violet (CV) staining assay was performed (O'Toole, 2011; Ren et al., 2014; Oliveira et al., 2015). Briefly, after removal of the supernatant, plates were washed twice with water, biofilms were stained with 1% CV for 10 min, plates were washed twice with water and air dried. To solubilize stained biofilm, 150 μl of 33% glacial acetic acid was added to each well, and the OD_595_ was measured.

### Co-culture Assays to Detect Contact-Dependent Interactions

The purpose of this part of the study was to test whether there were interactions between *Gardnerella* subgroups when they were grown together in the same culture. Four independent experiments were conducted at separate points in time with two different sets of *Gardnerella* isolates. Experiments 1A and 1B were done in March and April 2018, respectively, and subgroups A, B, C, and D were represented by isolates NR020, N170, NR038, and NR003. Experiments 2A and 2B were done in April and May 2018, respectively, and subgroups A, B, C, and D were represented by isolates VN003, VN002, NR001, and WP012. For each of the four replicate experiments, we grew the four subgroups alone (*n* = 4; A, B, C, D), and in all possible combinations of two (*n* = 6; AB, AC, AD, BC, BD, CD), three (*n* = 4; ABC, ABD, ACD, BCD), and four subgroups (*n* = 1; ABCD) for a total of 15 different combinations. Each of the 15 combinations was replicated three times in the wells of a 96-well tissue culture plate (i.e., 4 experiments ^*^ 15 combinations ^*^ 3 replicates per combination = 180 replicates). The members of each community were allowed to interact for a period of 48 h and the abundance of the constituent subgroups was estimated at the start (0 h) and the end (48 h) of this period using subgroup-specific quantitative real-time PCR (qPCR). Prior to the interaction assay, each of the four subgroups was grown alone at 37°C anaerobically in BHI with 0.25% maltose and 10% horse serum for a period of 12 h and then mixed in BHI + 0.25% maltose. Immediately prior to combining the subgroups to create the mixed communities, a sub-sample was taken from each of the four cultures to determine the abundance of each subgroup at the time point of 0 h using the subgroup-specific qPCR. To create the mixed communities, equal volumes of each isolate containing ~5 × 10^6^ genome equivalents per mL (i.e., verified by qPCR) were included in a total volume of 200 μl per well. For each of the four experiments, two plates were used: one for quantifying the number of cells in both the planktonic and biofilm fractions using qPCR and the other to quantify biofilm formation using the CV staining assay.

### Quantification of *Gardnerella* Using Subgroup-Specific Quantitative Real-Time PCR

Cells from planktonic and biofilm fractions were collected at 48 h and extraction of DNA was performed using a commercial kit (DNeasy PowerBiofilm, Qiagen, Mississauga, ON) following the manufacturer's instructions with minor modifications. To collect the planktonic phase, 200 μl of culture supernatant was removed from the wells and transferred to bead tubes. To collect biofilm, 200 μl of lysis reagent MBL was pipetted directly into the wells of the 96-well plate and incubated for 30 min to solubilize the biofilm. The bottom of each well was scraped with a pipette tip and the suspension was pipetted up and down several times before transferring it to a bead tube. The biofilm solubilization step was repeated to maximize biofilm collection. To enhance lysis, 100 μl of chaotropic agent FB was added to the bead tubes. The bead tubes were incubated at 65°C for 5 min in a water bath, and then vortexed using a multitube vortexer at maximum speed for 15 min. Later steps were performed following the manufacturer's instructions.

Subgroup-specific qPCR was performed using previously published primers and probes (Balashov et al., 2014) (Table S2). Amplification was performed in 10 μl reactions containing 1× iQ Supermix (BioRad, Mississauga, ON), 800 nM of each primer, 100 nM of TaqMan probe, and 2 μl of template. The qPCRs were performed using a CFX Connect (BioRad, Mississauga, ON) instrument. The qPCR results were reported as target copy number per PCR reaction (2 μl of template DNA extract). Each sample was assayed in duplicate reactions with the appropriate standard curve comprised of plasmids containing probe targets (10^2^-10^9^ plasmid copies per reaction). Thermocycling conditions were: initial denaturation at 95°C for 3 min, 40 cycles of 95°C for 15 s, and annealing/extension at 60°C (subgroups A, B, and D) or 63.3°C (subgroup C) for 40 s. Each plate contained a no template control, DNA extraction controls, and non-target subgroup templates as negative controls. For each qPCR reaction, the genome copy number was calculated using the standard curve. The qPCR assay was repeated for samples with a difference in Cq value > 1 between duplicate wells.

### Statistical Analysis of Contact-Independent Interactions

The contact-independent interactions were analyzed using Kruskal-Wallis non-parametric one-way ANOVA with Dunn's *post-hoc* test (Prism 8, Graphpad Software).

### Statistical Analysis of Contact-Dependent Interactions

**Figure 1:**
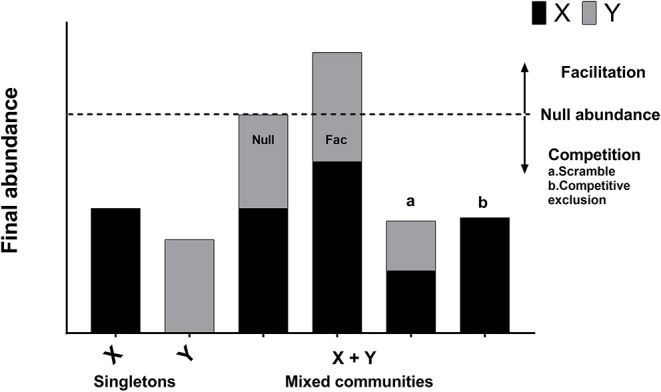
Regime of interpretation. Interactions between bacterial species or strains can be classified as neutral, competition, or facilitation. When bacterial species X and Y are grown in isolation, their abundances correspond to bars X and Y. Under the null hypothesis of no interaction, the expected abundance of the mixed community is equal to the sum of the abundances of two organisms grown separately (Null abundance, indicated by broken line). A neutral interaction occurs when the observed abundance of the mixed community is exactly equal to the null abundance (bar “Null”). In practice, neutral interactions are almost never observed due to measurement error. Facilitation occurs when the observed abundance of the mixed community is greater than the null abundance (bar “Fac”). Competition occurs when the observed abundance of the mixed community is less than the null abundance (bars “a” and “b”). Competitive exclusion occurs when one of the species is completely eliminated in the mixed community (bar “b”).

To characterize the interactions between the four subgroups, the 180 replicates of 15 unique communities in four independent experiments (1A, 1B, 2A, 2B) were analyzed collectively (4 experiments ^*^ 15 combinations per experiment ^*^ 3 replicates per combination = 180 replicates; each of the 15 unique combinations was replicated 12 times). Outcomes from these co-culture experiments were interpreted as shown in Figure 1 (Foster and Bell, 2012). First, the growth rates of all isolates grown as singletons were calculated using the following formula: r_i_ = ln (N_t, i_/N_0, i_)/T. Here, r_i_ is the observed growth rate of subgroup i when it is alone (has no competitors), N_0, i_ and N_t, i_ are the initial and final population sizes of subgroup i as estimated by qPCR (sum of biofilm and planktonic cells), and T is the time period of 48 h (Table S3). Under the null hypothesis of no competition, we used our estimates of r_i_ from the singletons and our estimates of N_0, i_ in the mixed communities to predict the expected abundance of each subgroup (${\hat{N}}_{t,\;i}$) in the mixed communities after 48 h of growth. The sum of predicted abundances (based on the singleton growth rates) for each subgroup in the community is the expected null abundance for the mixed communities (i.e., the expected community size in the absence of interaction). If the observed abundance of a mixed community was higher than the expected null abundance, the interaction was classified as facilitation (positive interaction). If the observed abundance of a mixed community was lower than the predicted null abundance, it was classified as competition (negative interaction) (Figure 1). The null hypothesis of no interaction predicts that due to random measurement error, 50% of the interactions should be positive (facilitation) and 50% of the interactions should be negative (competition). A proportion test was used to determine whether the observed prevalence of facilitation and competition were significantly different from the 50/50 expectation. This approach is a general test of the nature of interactions between *Gardnerella* subgroups and does not consider that each subgroup may be affected differently by competitors.

To test whether the subgroups were affected differently by the number of competitors, the growth rates of the *Gardnerella* subgroups were analyzed using linear mixed effect models (LMMs). The residuals of the growth rates were considered as normally distributed. The fixed effects were subgroup (four levels: A, B, C, and D), the number of competitors (0, 1, 2, and 3), and their interaction. The random effects were the 3 replicates of each community nested in the 4 different experiments (i.e., total of 12 replicates for each community). This approach does not consider the identity of the competitors. We used R (v 1.1-21) to analyze the data; the LMM models were run using the lmer() function in the R package lme4.

To test whether the identity of the competitors mattered, the growth rate of each subgroup was analyzed separately using LMMs. The four subgroups had to be analyzed separately, because the identity of the competitors differs for each subgroup. The fixed effects were the identities of the competitors. For example, for the growth rate of subgroup A, the competitors included B, C, D, BC, BD, CD, and BCD. The random effects structure was the same as before.

## Results

### Effect of *Gardnerella* Culture Supernatant on Growth and Biofilm Formation

The initial optical density (OD) of all the focal strains was ~0.05 and they grew in both NYC III and BHI + 1% glucose, except for one subgroup C strain, NR001, which did not grow in BHI + 1% glucose (Figure S11). The OD at 48 h of these strains varied from as low as 0.05 (after subtracting initial OD) to 0.80. There was no effect of CFS on overall growth or planktonic growth of focal strains, nor on biofilm formation (Kruskal-Wallis non-parametric one-way ANOVA with Dunn's *post-hoc* test, *p* > 0.05 for all comparisons) (Figures S1–S14). The type of medium, however, influenced mode of growth: NYC III had more planktonic growth, whereas BHI + 1% glucose had more biofilm growth. Increasing the concentration of CFS from 10 to 20% had no effect on the growth pattern of the *Gardnerella* subgroups (data not shown).

### Validation of qPCR Assays

Prior to performing the co-culture experiments, the efficiency of each subgroup-specific qPCR assay and the limits of detection and quantification were determined, since these values had not been reported previously (Balashov et al., 2014). The amplification efficiency for subgroups A, B, C, and D were 99.9, 107.4, 110, and 98.2%, respectively. The lowest concentration at which all subgroups were detected was 1 target copy per qPCR reaction. However, the lower limit of quantification (LOQ) was different for each subgroup. The LOQ for subgroups A, B, C, and D were 1, 10, 100, and 1 copy per reaction, respectively.

### Characterization of Contact-Dependent Interactions Between *Gardnerella* Isolates

Our null hypothesis approach of testing the type of interaction (facilitation vs. competition) between subgroups of *Gardnerella* found that competition was 2.3 times more common than facilitation. Of the 132 mixed communities, 69.7% (92/132) had negative interactions (competition), and 30.3% (40/192) had positive interactions (facilitation) (Table S3). A proportion test found that these percentages were significantly different (*p* < 0.0001) from the 50/50 null expectation. Competition was more frequently observed in communities with more subgroups. The prevalence of competition was 58.3% (42/72), 79.2% (38/48), and 100.0% (12/12) in communities with two, three, or four subgroups, respectively.

Alternatively, we can set an arbitrary threshold so that absolute differences <500 million cells (between the observed and expected values) are considered as neutral interactions (i.e., the difference was caused by random measurement error). Using this approach for the 132 mixed communities, there were 25.0% (33/132) neutral interactions, 75.0% (99/132) competitive interactions, and 0.0% (0/132) facilitative interactions. The probability of getting this result under the null hypothesis that competition and facilitation are equally likely (50/50) is vanishingly small (*p* < 0.0001). This alternative analysis shows that introducing an arbitrary threshold to separate measurement error from biologically interesting interactions does not change our conclusion that competitive interactions dominate between *Gardnerella* subgroups.

### Competition Between Subgroups in Biofilms vs. the Supernatant

Since biofilms are a common site of interactions between species (Nadell et al., 2009; Elias and Banin, 2012; Burmølle et al., 2014; Oliveira et al., 2015), we investigated whether competition was more frequent in the biofilm fraction than the planktonic fraction. Out of 132 mixed communities, 68.9% of the biofilm fractions (91/132), and 65.9% (87/132) of the planktonic fractions exhibited competition (Table S3). This result indicates that competition occurs in both biofilm and planktonic populations of *Gardnerella* spp. In addition, these observations demonstrate that mixed subgroup biofilms can occur, with no subgroup excluded.

### Effect of Mixed Communities on the Growth Rate of Individual Subgroups

**Figure 2:**
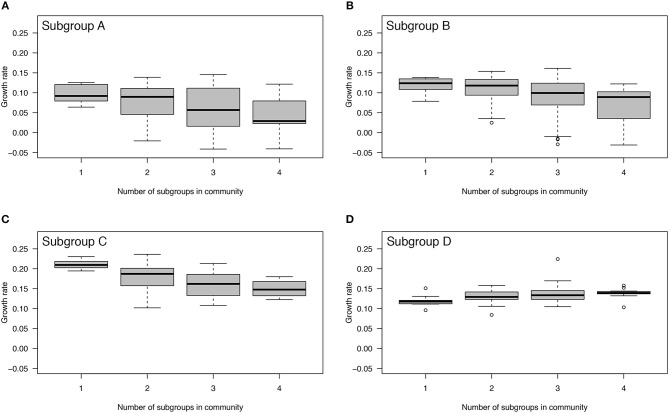
Effect of mixed communities on growth rates of *Gardnerella* subgroups. The growth rates of subgroups A, B, and C decreased with an increasing number of competitors indicating competition **(A–C)**. In contrast, the growth rate of subgroup D increased with an increasing number of competitors indicating facilitation **(D)**. Each community was allowed to interact and grow over a period of 48 h.

When grown in isolation, the instantaneous growth rates (per hour) of the four subgroups ranked from lowest to highest are as follows: 0.098 for subgroup A, 0.119 for subgroup D, 0.120 for subgroup B, and 0.211 for subgroup C. The population doubling times (in hours) ranked from slowest to fastest are as follows: 7.1 for subgroup A, 5.8 for subgroup D, 5.8 for subgroup B, and 3.3 for subgroup C. The LMM found a significant interaction between subgroup and number of competitors on the growth rate (*p* < 0.0001) indicating that the effect of the number of competitors on the growth rate differs between subgroups. Growth rates of subgroups A, B, and C decreased significantly (*p* < 0.0001) in mixed communities (Figures 2A–C). In contrast, the growth rate of subgroup D increased significantly (*p* < 0.0001) in mixed communities (Figure 2D). Thus, subgroups A, B, and C experienced competition in mixed communities, whereas subgroup D experienced facilitation. Regardless of the identity of the community, subgroup C always had a higher intrinsic growth rate than the other subgroups (Figure 2).

### Impact of Competitor Subgroups on Focal Subgroups

**Figure 3:**
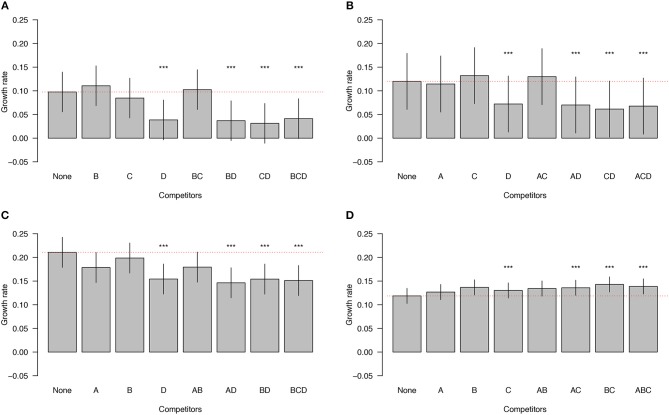
Impact of competitor subgroups on focal subgroup. The growth rates of subgroups A, B, and C are inhibited by the presence of subgroup D **(A–C)**. In contrast, the growth rate of subgroup D is enhanced by the presence of the other subgroups **(D)**. The identities of the competitors are indicated on the X axis. The red dotted line is the expected null growth rate (growth as a singleton). Growth rates above and below the red dotted line indicate facilitation and competition, respectively. Significant effects on growth rates of the focal subgroups in the presence of competitors are denoted by asterisks (^***^). The vertical lines on each bar indicate the 95% confidence intervals for the means.

Next, we investigated whether subgroups differed in the magnitude of their negative (or positive) effect on the growth rate of other subgroups. Subgroup D had the most negative impact on the growth rates of the other subgroups. The presence of subgroup D in any community reduced the growth rate of the other members of the communities by 44.2% (Figures 3A–C; LMM, *p* < 0.0001). Subgroup A reduced the growth rates of the other members of the communities by 4.8%, whereas B and C increased the growth rates of the other members of the communities by 7.2%, and 1.6%, but none of these effects were statistically significant (Figure 3). In summary, subgroup D has a large and negative effect on the growth rate of all other subgroups, whereas the effects of the other subgroups are essentially neutral.

### Quantification of Biofilm Formation in Monocultures and Co-cultures

**Figure 4:**
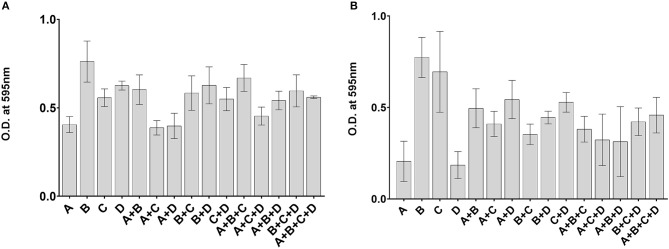
No enhancement of biofilm formation in mixed communities. Isolates used in Experiment 1 **(A)** and Experiment 2 **(B)** were cultured alone or in combinations. Biofilm biomass was quantified by a crystal violet assay after 48 h of growth. The error bars indicate standard deviations of three replicates.

Mixing of different bacterial species often leads to increased biofilm formation. We therefore investigated whether mixing of *Gardnerella* subgroups would enhance biofilm formation. If the amount of biofilm formed by a mixture of subgroups was greater than the amount formed by the best individual biofilm former of that mixture, the interaction was considered synergistic. If the amount of biofilm formed by a mixture was less than the amount formed by the worst individual biofilm former of that mixture, the interaction was considered antagonistic (Madsen et al., 2016). Here biofilm formation by mixed subgroups was almost always less than the individual biofilm formation by the best biofilm-forming subgroup but greater than the worst biofilm former in the mixture (Figures 4A,B). Only one co-culture of subgroups A and D was higher than the individual biofilm formation of either strain (Kruskal-Wallis, *p* < 0.05) (Figure 4B). The results of this experiment show that overall biofilm biomass is not enhanced by mixing of subgroups.

### Effect of *Gardnerella* Co-culture Supernatant on Individual Subgroups

Our initial experiments showed that the CFS of singleton cultures had no impact on the growth of isolates from other subgroups, but negative interactions were frequently observed in co-cultures. To determine if effectors were secreted as a result of contact, we derived CFS from pairwise co-cultures and prepared media conditioned with 10% co-culture supernatant. We grew four representative isolates of all four subgroups in media with and without co-culture CFS and measured optical density to monitor growth and used a CV assay for quantification of biofilm formation (Figure S15). No significant differences in the amount or mode of growth were observed with exposure to co-culture CFS (Kruskal-Wallis, *p* > 0.05).

## Discussion

A critical step in the development of bacterial vaginosis is when *Gardnerella* spp. displace *Lactobacillus* spp. and initiate multispecies biofilm formation (Schwebke et al., 2014). The recent amendment of the genus and the “fine-tuning” of the taxonomy of *Gardnerella* (Vaneechoutte et al., 2019) re-emphasizes the clinical importance of determining the particular contributions of different *Gardnerella* spp. to vaginosis, and the degree to which different subgroups may compete or cooperate in the vaginal microbiome. The detection of multiple subgroups (or species) of *Gardnerella* in the vaginal microbiomes of individual women is common and so interactions are expected to occur frequently (Hardy et al., 2016, 2017; Castro et al., 2019). Our current study was designed to determine the types of interactions that occur between isolates from different cpn60-defined subgroups of *Gardnerella*, and to discover whether multiple subgroups can be incorporated into biofilms.

### No Evidence of Contest Competition Between Subgroups of *Gardnerella*

A contest is a direct, interference competition where the secretion of small molecules (secondary metabolites or toxins) by one organism inhibits the growth of other organisms in an environment (Czárán et al., 2002; Hibbing et al., 2010; Faust and Raes, 2012; Garbeva et al., 2014; Oliveira et al., 2015; Ismail et al., 2016; Stubbendieck and Straight, 2016). Cell-free supernatant (CFS) is the first place to look for any such secreted small molecules that could affect the growth of other bacterial species or strains. *Gardnerella* isolates have been shown to inhibit the growth of some vaginal lactobacilli in a contact-independent manner (McLean and McGroarty, 1996; Teixeira et al., 2010), and both inhibitory and stimulatory effects of *Gardnerella* CFS on the growth of a range of vaginal microbiota have been documented (Chanos and Mygind, 2016). These previous reports, however, have involved relatively few isolates and no information was provided regarding the *Gardnerella* species. In the present study, we detected no effect on the amount or mode of growth of *Gardnerella* isolates when they were exposed to the CFS from other isolates grown in isolation (Figures S1–S14). Since effector molecules are often only secreted when their producers are in contact with other bacterial species (Konovalova and Søgaard-Andersen, 2011; Chanos and Mygind, 2016), we also tested whether CFS from co-culture combinations (where competition had been observed in co-culture assays) affected the growth of *Gardnerella* strains. We found no effect of co-culture CFS on growth, which further supports the conclusion that there is no contest or direct interference competition between *Gardnerella* subgroups (Figure S15). Similarly, no enhancement of growth was observed, which would have been expected if there was nutritional synergy or cross-feeding among *Gardnerella* spp. as has been demonstrated for *G. vaginalis* and *Prevotella bivia* (Pybus and Onderdonk, 1997).

### Scramble Competition Is Common in Mixed Communities of *Gardnerella*

When *Gardnerella* isolates from different subgroups were co-cultured, all of them were present in both planktonic and biofilm fractions of each tested community, indicating that no subgroup was completely dominant or excluded over the 48-h observation period. Competition between subgroups was common, with 70% of the observed interactions classified as competitive. Although intrinsic growth rates differed among the four subgroups (Figure 2), subgroups A, B and C all showed a reduced growth rate as the number of competitors increased (Figures 2A–C). Interestingly, subgroup D experienced facilitation in co-cultures because its growth rate increased with increasing numbers of competitors (Figure 2D). Subgroup D also had a negative effect on the growth rates of other subgroups (Figures 3A–D). Taken together, these co-culture observations are consistent with a non-interfering, exploitative competition, which is also called scramble competition (Nicholson, 1954; Hibbing et al., 2010). Scramble competitions result in the dominance of the competitor with the greatest ability to exploit a shared resource (e.g., nutrients), and a general reduction in the overall fitness of all members of a mixed community that share this resource (Darwin, 1859; Nicholson, 1954; Foster and Bell, 2012).

One possible explanation for the distinct behavior of subgroup D is that it has different nutritional requirements than the other subgroups and thus remains unaffected when others compete for the same nutrient resources. It might also represent a “social cheater” (Hibbing et al., 2010; Ghoul et al., 2014); an opportunistic member of the community that occupies a distinct niche and benefits from the competition of others. Subgroup D strains are rarely detected in the vaginal microbiome, and are usually at low abundance (Albert et al., 2015). Negative-frequency dependent selection can favor rare variants that are able to exploit available niches (Hibbing et al., 2010), allowing them to thrive in an otherwise highly competitive environment (Ghoul et al., 2014). These specialized variants can also make some important nutrients unavailable to the other community members, who are carrying the cost of maintaining the multispecies community (Harrison et al., 2006). This scenario could explain why, in mixed *Gardnerella* subgroup communities *in vitro*, the presence of subgroup D negatively affects the growth of other subgroups. *In vivo*, the interactions between other bacterial species in the vaginal microbiome might check the abundance of this social cheater (Hibbing et al., 2010). Since our experiments were conducted over a relatively short period of time (48 h), we were unable to determine if mixed communities of *Gardnerella* subgroups comprise a non-transitive competitive interaction network. This type of interaction is characterized by gradual replacement of dominant species by others in the consortium (Hibbing et al., 2010), but requires the presence of other contributing factors and changes in the environment happening over time that could not be captured in our *in vitro* model. A longitudinal study using a dynamic culture system might possibly demonstrate the presence of a non-transitive network in *Gardnerella* spp.

### No Synergy in Mixed Subgroup Biofilm

*Gardnerella* species have been implicated in the initiation of vaginal biofilms by displacing lactobacilli and adhering to the epithelium. Subsequent recruitment of other bacteria results in the characteristic multispecies bacterial vaginosis biofilm (Machado and Cerca, 2015; Hardy et al., 2016; Castro et al., 2019). Multispecies biofilms are a hotspot of interactions (Burmølle et al., 2014; Liu et al., 2016) that can be antagonistic or synergistic (Ren et al., 2015; Røder et al., 2015). Synergy can result in increased biofilm biomass in co-cultures compared to the best individual biofilm former grown alone, while antagonism can lead to a reduction in the biofilm biomass of co-culture compared to the worst individual biofilm former (Madsen et al., 2016). Enhancement of biofilm formation can also be the result of competition where the end result is the exclusion of some species from the biofilm (Foster and Bell, 2012; Oliveira et al., 2015). In the current study, no enhancement of biofilm biomass was detected using a CV assay when different *Gardnerella* subgroups were co-cultured (Figure 4), which is consistent with the non-interfering, exploitative competition we observed in the co-cultures. Importantly, our results show that all subgroups of *Gardnerella* can participate in biofilms, and thus contribute to the formation of this defining feature of bacterial vaginosis, regardless of their individual arsenals of “virulence factors.”

## Limitations

The logistical advantages of *in vitro* systems for studying bacterial interactions are balanced by some limitations. We used a closed system with one set of growth conditions where nutrient depletion over the duration of the experiments may have affected interactions. In addition, the *in vitro* environment lacks the other members of the vaginal microbiota, host immune system, vaginal fluid flow, epithelial cell turnover, and other environmental cues present in the vaginal ecosystem. In the absence of an appropriate animal model, microfluidic devices, and cultured vaginal epithelial cells may offer more realistic conditions, but also present logistical challenges for studies involving large numbers of isolates and experimental replication. In our experiments, *Gardnerella* cell numbers were estimated based on quantitative PCR, which does not distinguish viable from non-viable bacteria, but since we used growth rate as our main measurement, this factor was likely not a major influence on our results. Another potential limitation of the study is the isolates we used were chosen to represent *Gardnerella* subgroups, and were not isolated as pre-established consortia from individual women. It has been proposed that *Gardnerella* biofilms can be sexually transmitted (Swidsinski et al., 2014), and since our results demonstrate the possibility of biofilms comprised of multiple *Gardnerella* spp., it will be interesting to investigate interactions among isolates from biofilms that may have been transmitted together over long periods of time.

## Conclusions

Overall, our experiments suggest that competition is common in mixed communities of *Gardnerella* subgroups and that these negative interactions are likely due to niche overlap and competition for shared resources rather than direct interference. The combined effects of scramble competition and different vaginal microbiota compositions in individual women, physiological influences, medical interventions, and sexual and hygiene practices, results in the patterns of distribution of *Gardnerella* spp. we observe in reproductive-aged women. Colonization by multiple species is common and any one of the most frequently detected subgroups (A, B, and C, corresponding to *G. swidsinskii, leopoldii, piotii*, and *vaginalis*) can dominate the microbiome. Longitudinal studies of *Gardnerella* spp. in co-culture will be critical in deciphering the contributions of both abundant and rare species in the transition to bacterial vaginosis in the vaginal microbiome.

## Data Availability Statement

All datasets generated for this study are included in the article/Supplementary Material.

## Author Contributions

SK and JH conceived and designed the study. SK performed the experiments and wrote the first draft of the manuscript. MV and SK conducted the statistical analyses. All authors contributed to manuscript revision, read, and approved the submitted version.

### Conflict of Interest

The authors declare that the research was conducted in the absence of any commercial or financial relationships that could be construed as a potential conflict of interest.
