# Supplementary material for: Competition Among Gardnerella Subgroups From the Human Vaginal Microbiome
Source: Front Cell Infect Microbiol. 2019 Oct 31;9:374. doi: 10.3389/fcimb.2019.00374 (PMC6834547; doi:10.3389/fcimb.2019.00374)
Supplement: Supplementary file 1 [file Data_Sheet_1.pdf]

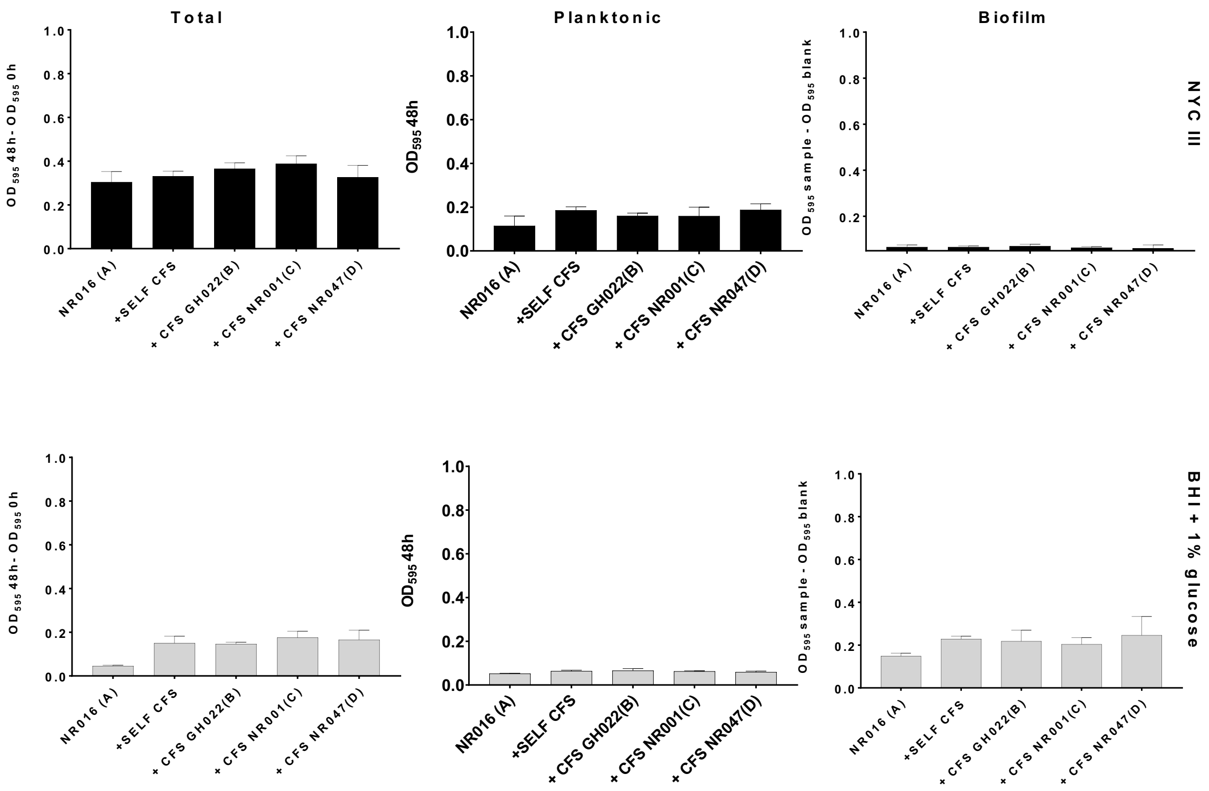

Fig S1: Growth of NR016 (subgroup A) in two different media: NYC III (upper panels, black bars) and BHI with 1% glucose (lower panels, grey bars). NYC III and BHI were spiked with 10% self-CFS, CFS of GH022 (subgroup B), NR001 (subgroup C) and NR047 (subgroup D) to challenge NR016. Total growth, planktonic growth and biofilm formation were measured at 48 h as described in the text. Error bars represent standard deviations of triplicates.

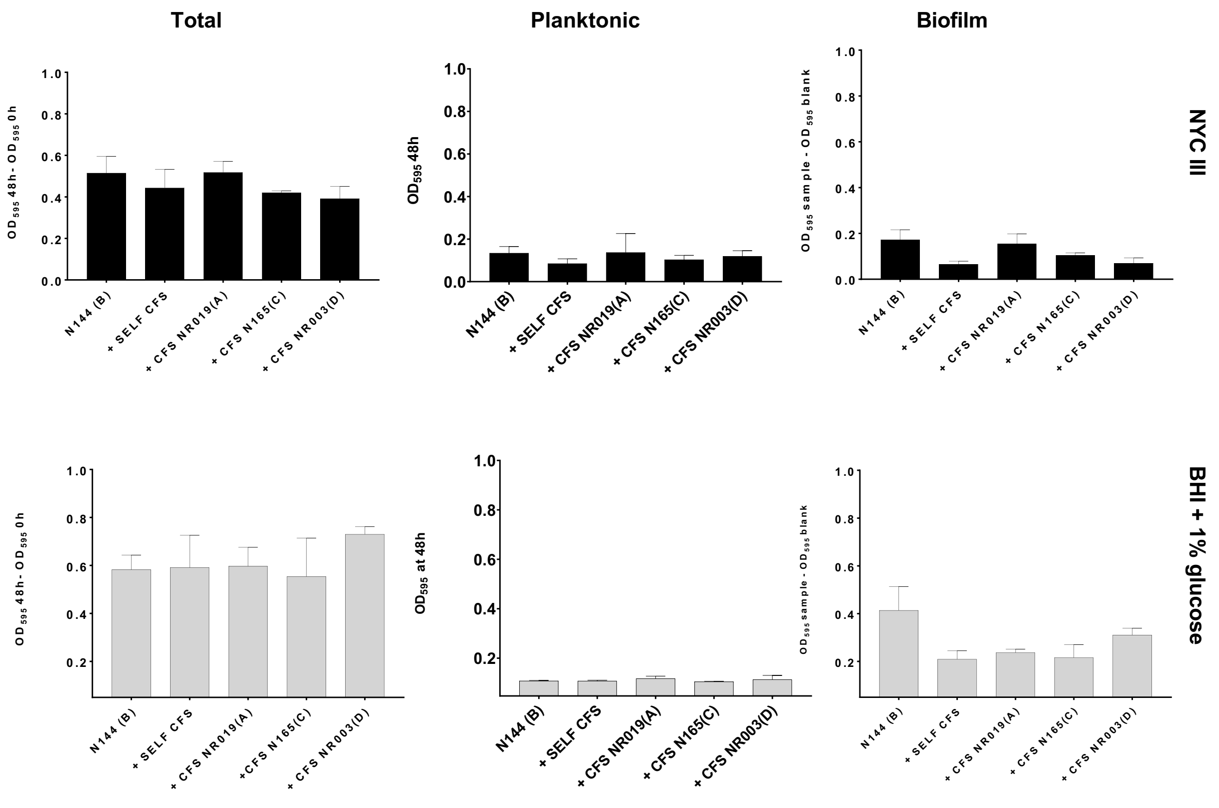

Fig S2: Growth of N144 (subgroup B) in two different media: NYC III (upper panels, black bars) and BHI with 1% glucose (lower panels, grey bars). NYC III and BHI were spiked with 10% self-CFS, CFS of NR019 (subgroup A), N165 (subgroup C) and NR003 (subgroup D) to challenge N144. Total growth, planktonic growth and biofilm formation were measured at 48 h as described in the text. Error bars represent standard deviations of triplicates.

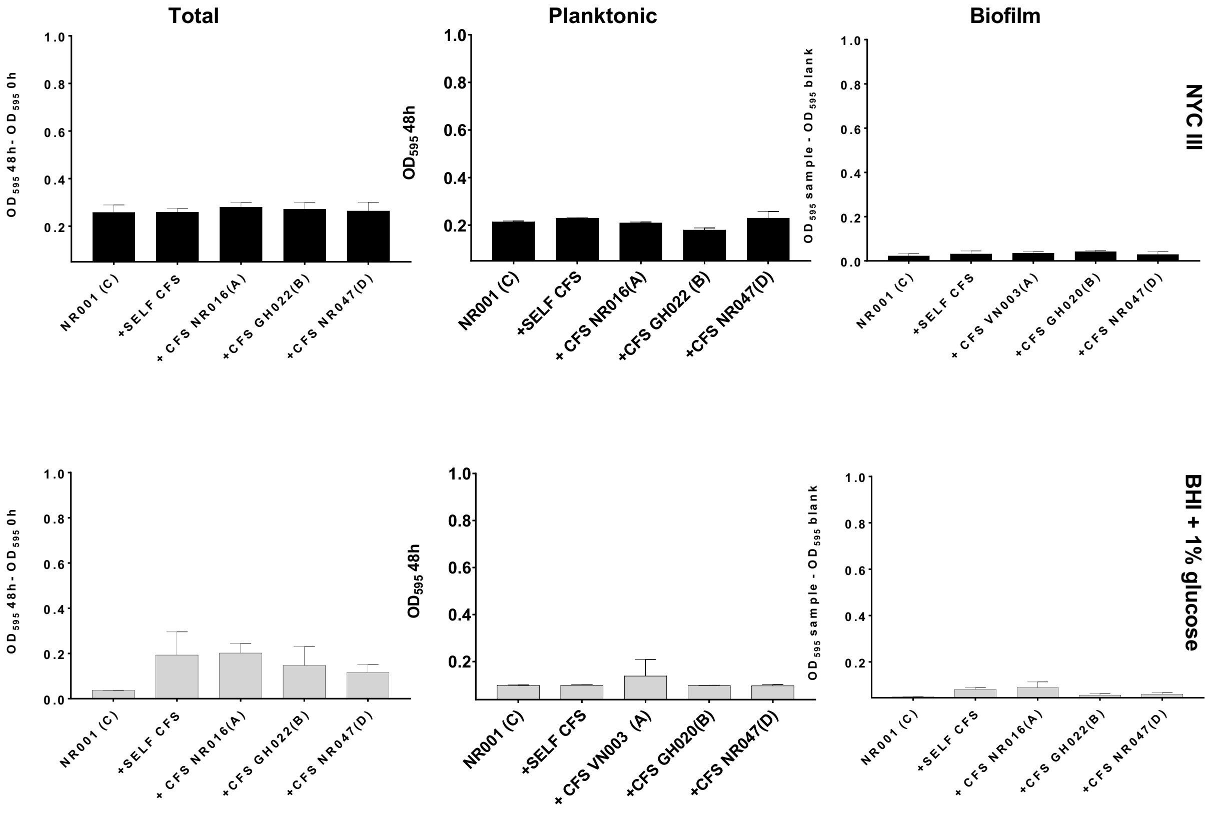

Fig S3: Growth of NR001 (subgroup C) in two different media: NYC III (upper panels, black bars) and BHI with 1% glucose (lower panels, grey bars). NYC III and BHI were spiked with 10% self-CFS, CFS of VN003 (subgroup A), GH020 (subgroup B) and NR047 (subgroup D) to challenge NR047. Total growth, planktonic growth and biofilm formation were measured at 48 h as described in the text. Error bars represent standard deviations of triplicates.

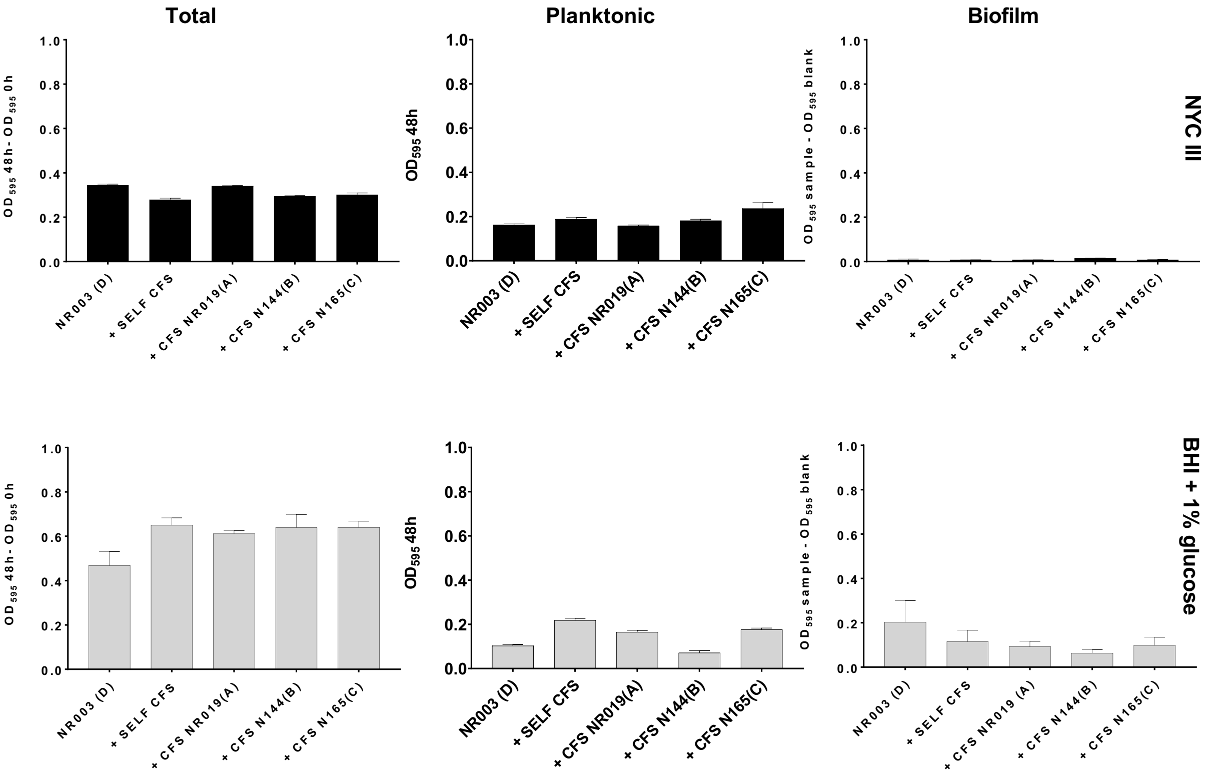

Fig S4: Growth of NR003 (subgroup D) in two different media: NYC III (upper panels, black bars) and BHI with 1% glucose (lower panels, grey bars). NYC III and BHI were spiked with 10% self-CFS, CFS of NR019 (subgroup B), N144 (subgroup B) and N165 (subgroup C) to challenge NR016. Total growth, planktonic growth and biofilm formation were measured at 48 h as described in the text. Error bars represent standard deviations of triplicates.

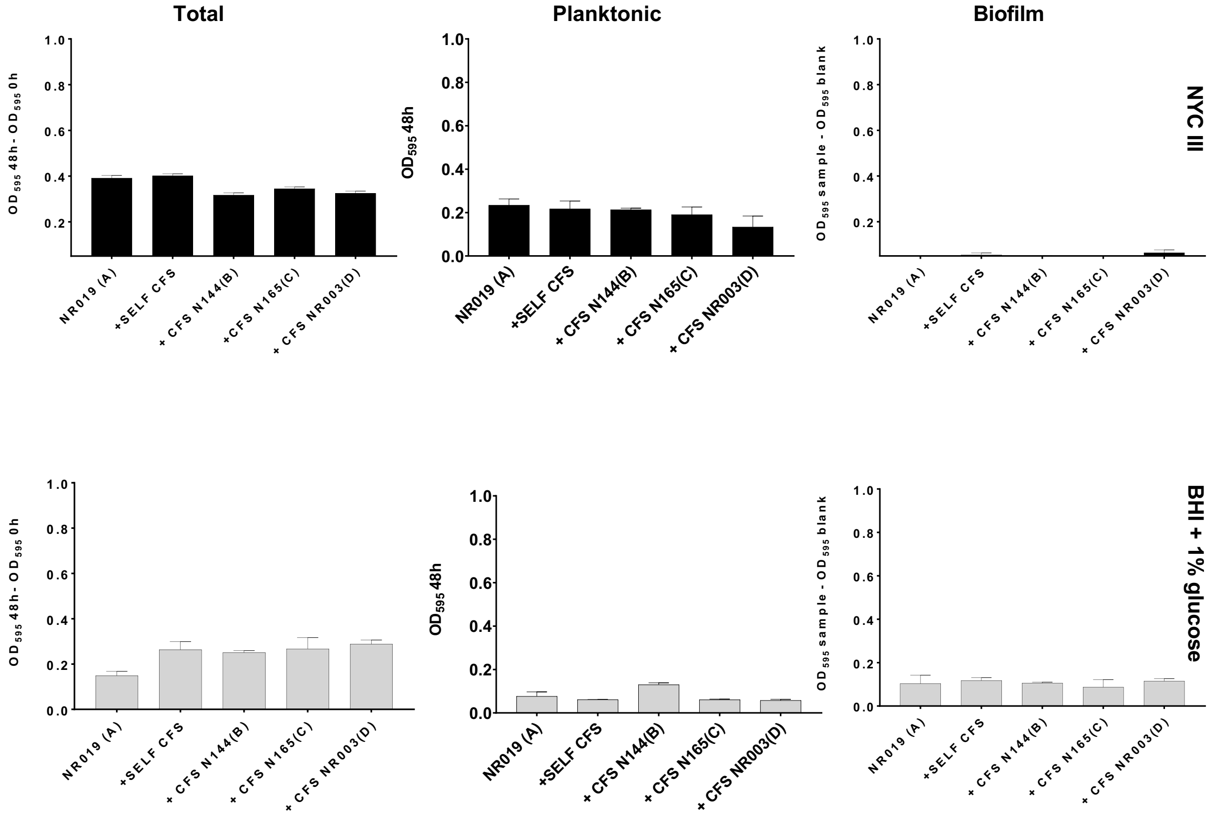

Fig S5: Growth of NR019 (subgroup A) in two different media: NYC III (upper panels, black bars) and BHI with 1% glucose (lower panels, grey bars). NYC III and BHI were spiked with 10% self-CFS, CFS of N144 (subgroup B), N165 (subgroup C) and NR003 (subgroup D) to challenge NR019. Total growth, planktonic growth and biofilm formation were measured at 48 h as described in the text. Error bars represent standard deviations of triplicates.

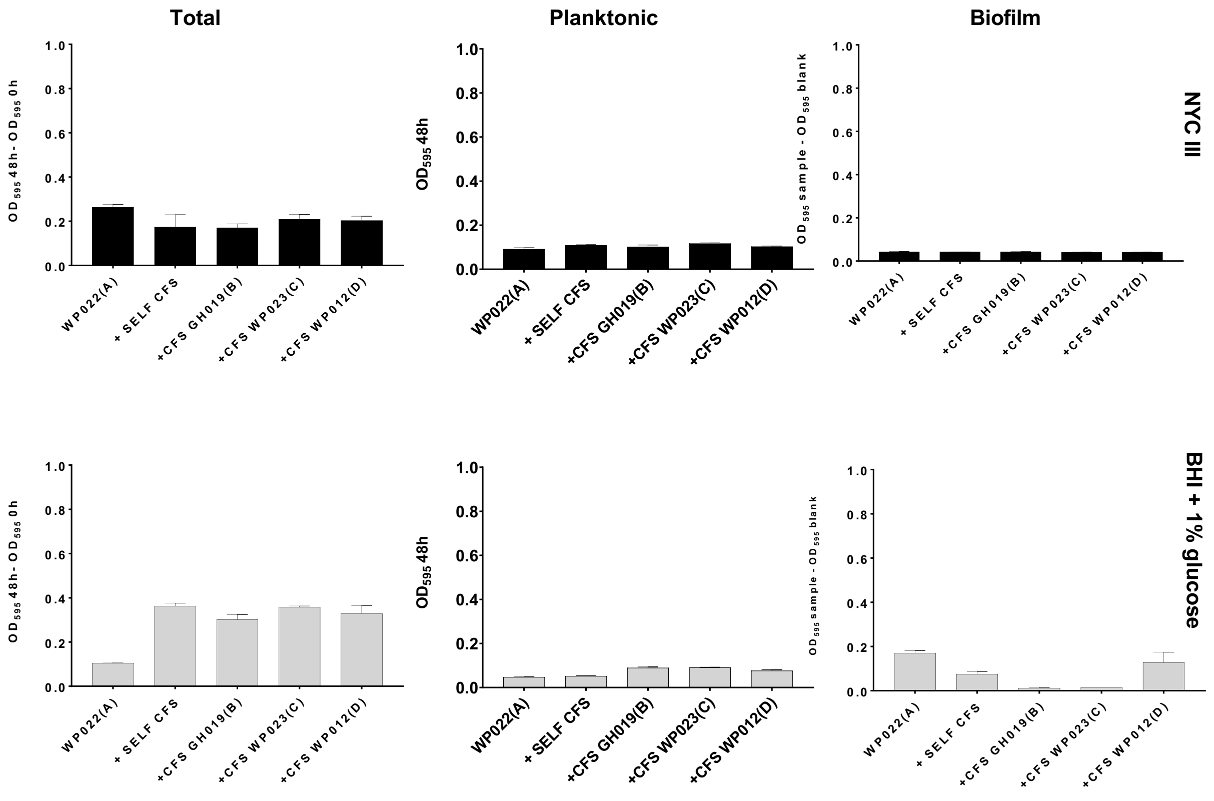

Fig S6: Growth of WP022 (subgroup A) in two different media: NYC III (upper panels, black bars) and BHI with 1% glucose (lower panels, grey bars). NYC III and BHI were spiked with 10% self-CFS, CFS of GH019 (subgroup B), WP023 (subgroup C) and WP012 (subgroup D) to challenge NR016. Total growth, planktonic growth and biofilm formation were measured at 48 h as described in the text. Error bars represent standard deviations of triplicates.

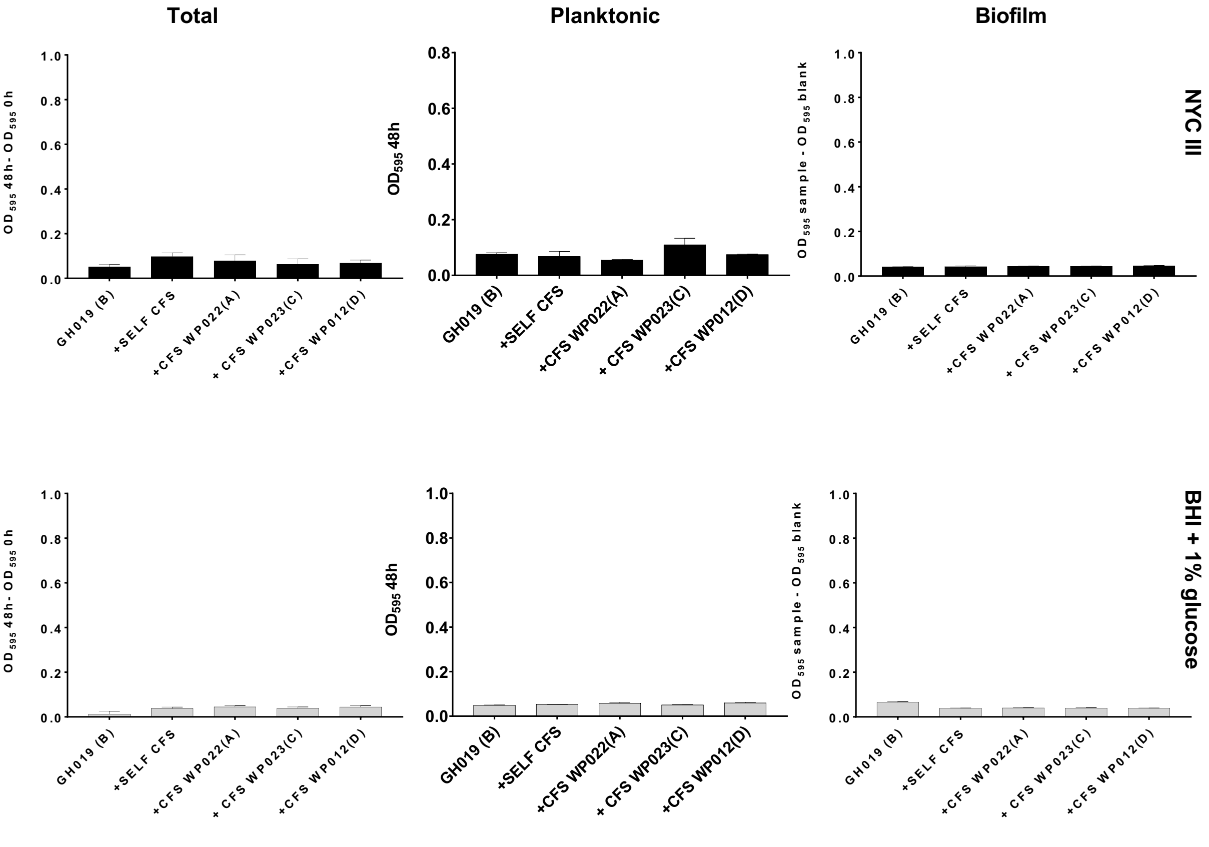

Fig S7: Growth of GH019 (subgroup A) in two different media: NYC III (upper panels, black bars) and BHI with 1% glucose (lower panels, grey bars). NYC III and BHI were spiked with 10% self-CFS, CFS of WP022(subgroup A), WP023 (subgroup C) and WP012 (subgroup D) to challenge GH019. Total growth, planktonic growth and biofilm formation were measured at 48 h as described in the text. Error bars represent standard deviations of triplicates.

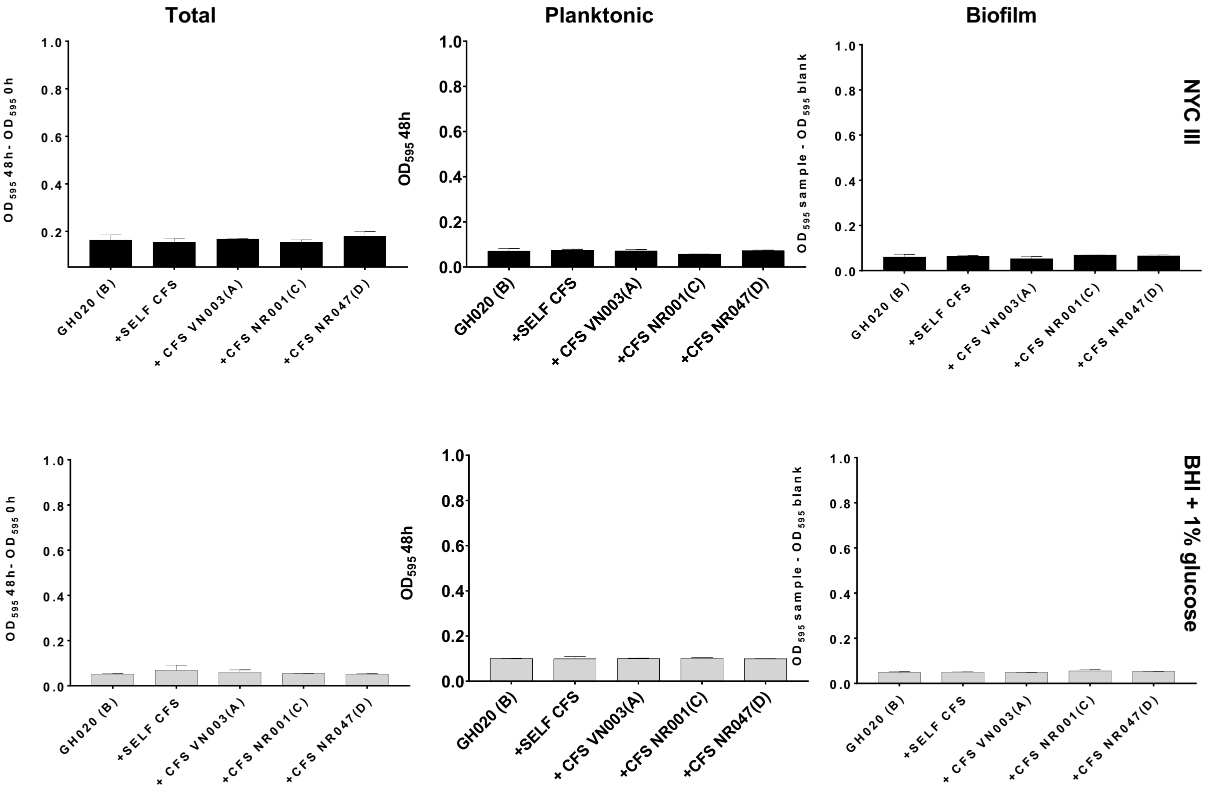

Fig S8: Growth of GH020 (subgroup A) in two different media: NYC III (upper panels, black bars) and BHI with 1% glucose (lower panels, grey bars). NYC III and BHI were spiked with 10% self-CFS, CFS of VN003 (subgroup A), NR001 (subgroup C) and NR047 (subgroup D) to challenge NR016. Total growth, planktonic growth and biofilm formation were measured at 48 h as described in the text. Error bars represent standard deviations of triplicates.

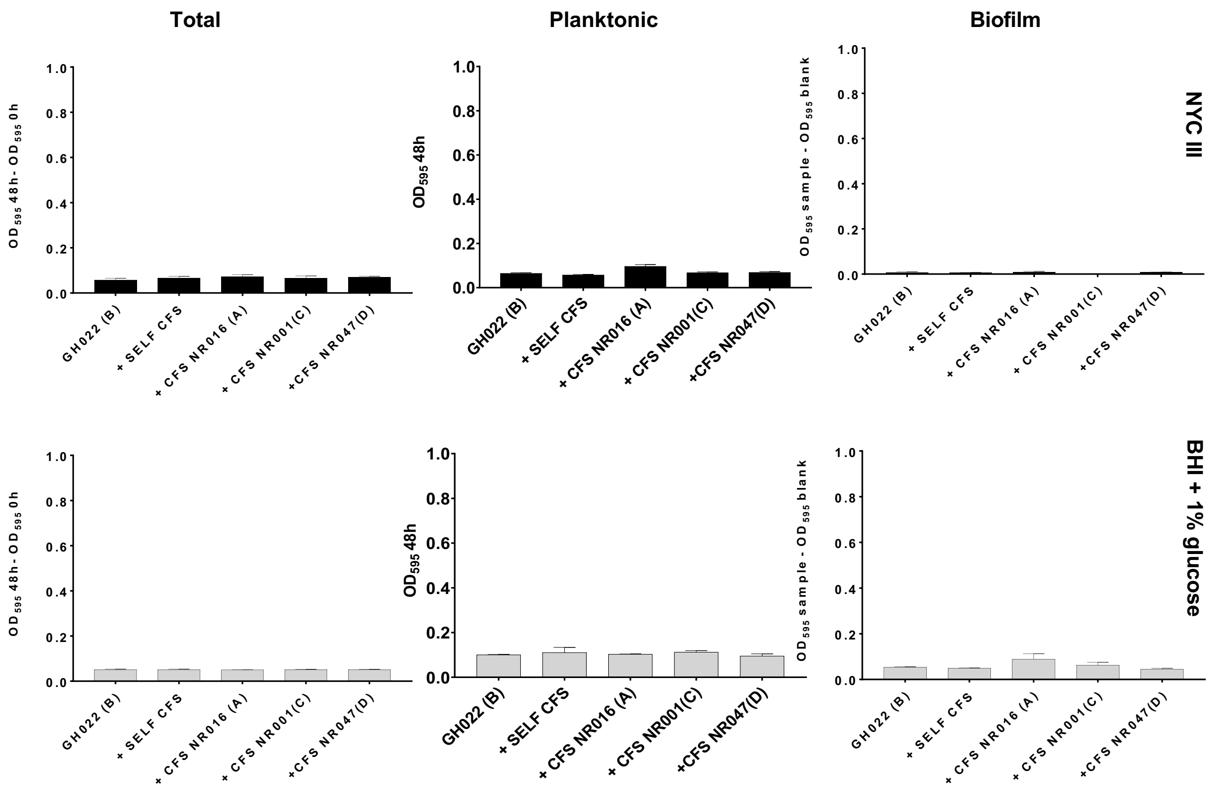

Fig S9: Growth of GH022 (subgroup B) in two different media: NYC III (upper panels, black bars) and BHI with 1% glucose (lower panels, grey bars). NYC III and BHI were spiked with 10% self-CFS, CFS of NR016(subgroup A), NR001 (subgroup C) and NR047 (subgroup D) to challenge NR016. Total growth, planktonic growth and biofilm formation were measured at 48 h as described in the text. Error bars represent standard deviations of triplicates.

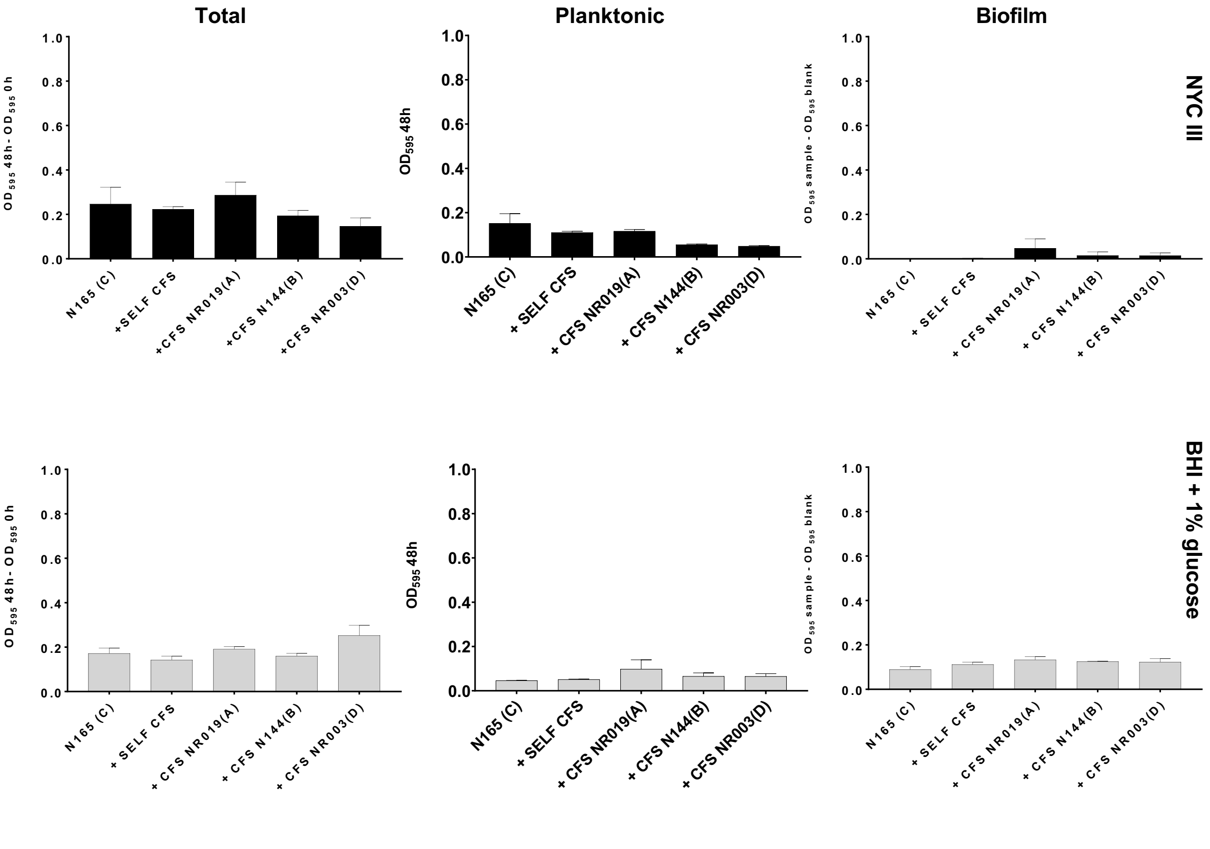

Fig S10: Growth of N165 (subgroup C) in two different media: NYC III (upper panels, black bars) and BHI with 1% glucose (lower panels, grey bars). NYC III and BHI were spiked with 10% self-CFS, CFS of NR019 (subgroup A), N144 (subgroup B) and NR003 (subgroup D) to challenge N165. Total growth, planktonic growth and biofilm formation were measured at 48 h as described in the text. Error bars represent standard deviations of triplicates.

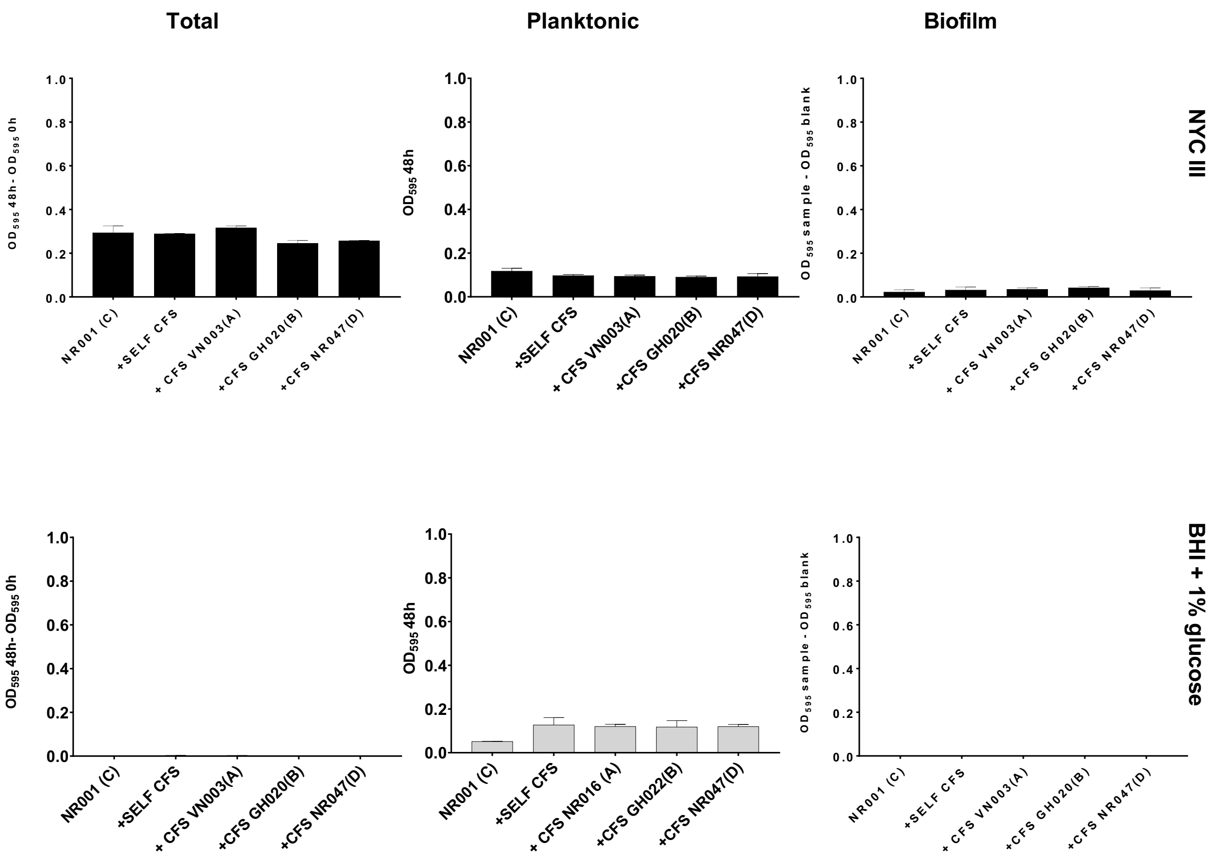

Fig S11: Growth of NR001 (subgroup C) in two different media: NYC III (upper panels, black bars) and BHI with 1% glucose (lower panels, grey bars). NYC III and BHI were spiked with 10% self-CFS, CFS of VN003 (subgroup A), NR001 (subgroup C) and NR047 (subgroup D) to challenge NR001. Total growth, planktonic growth and biofilm formation were measured at 48 h as described in the text. Error bars represent standard deviations of triplicates.

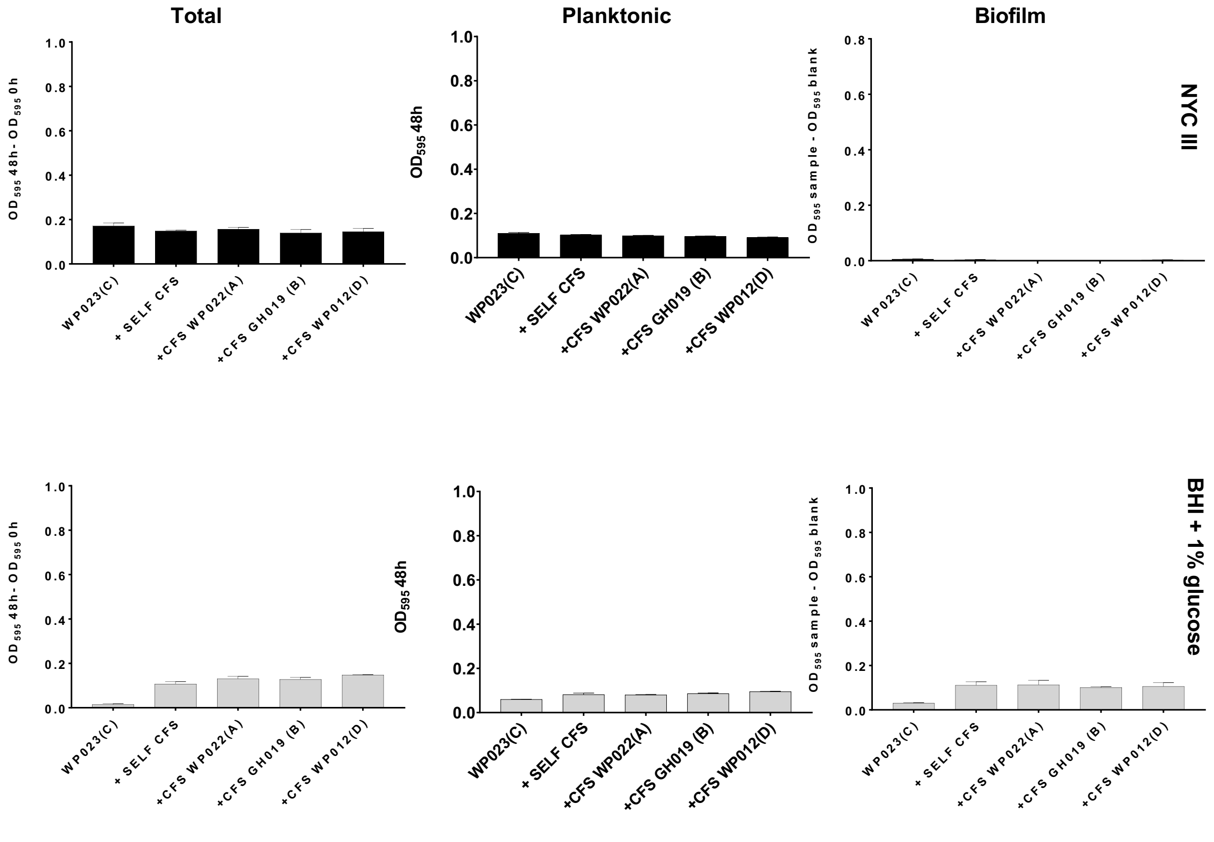

Fig S12: Growth of WP023 (subgroup C) in two different media: NYC III (upper panels, black bars) and BHI with 1% glucose (lower panels, grey bars). NYC III and BHI were spiked with 10% self-CFS, CFS of WP022 (subgroup A), GH019 (subgroup B) and WP012 (subgroup D) to challenge WP023. Total growth, planktonic growth and biofilm formation were measured at 48 h as described in the text. Error bars represent standard deviations of triplicates.

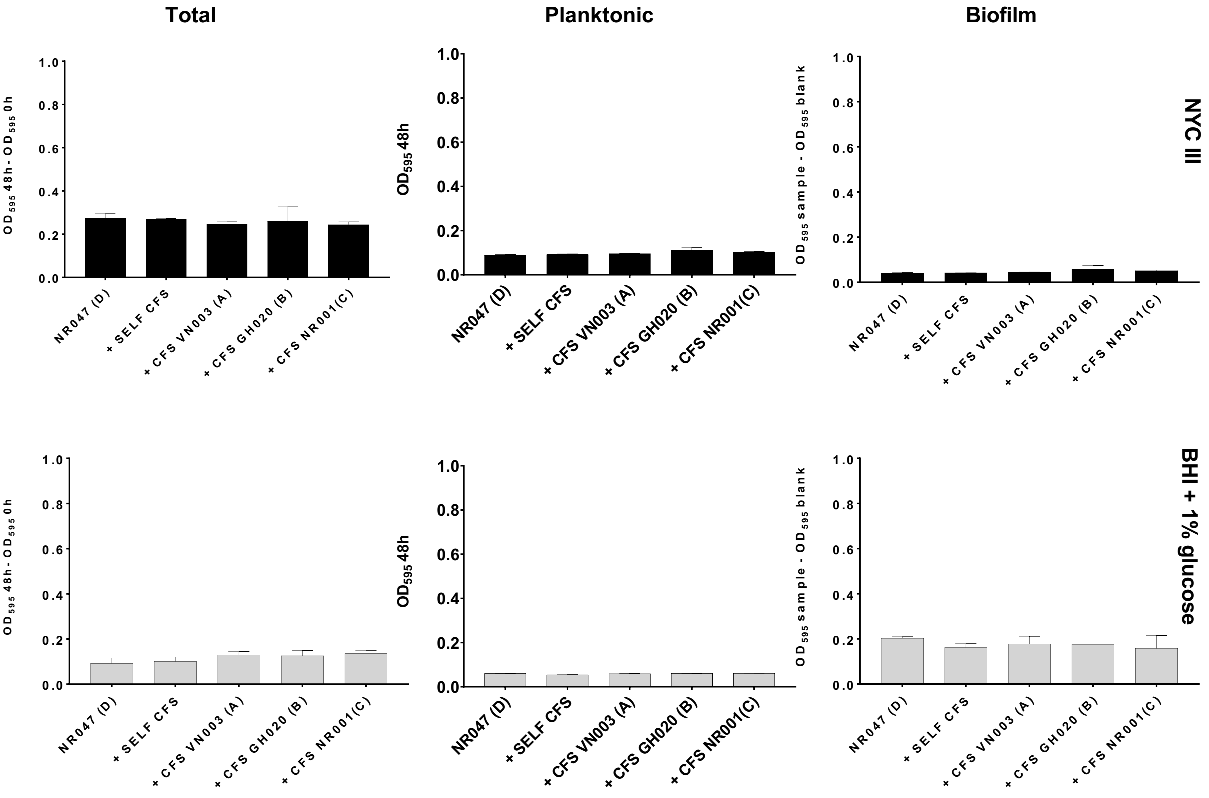

Fig S13: Growth of NR047 (subgroup D) in two different media: NYC III (upper panels, black bars) and BHI with 1% glucose (lower panels, grey bars). NYC III and BHI were spiked with 10% self-CFS, CFS of GH020 (subgroup B), NR001 (subgroup C) and NR047 (subgroup D) to challenge NR047. Total growth, planktonic growth and biofilm formation were measured at 48 h as described in the text. Error bars represent standard deviations of triplicates.

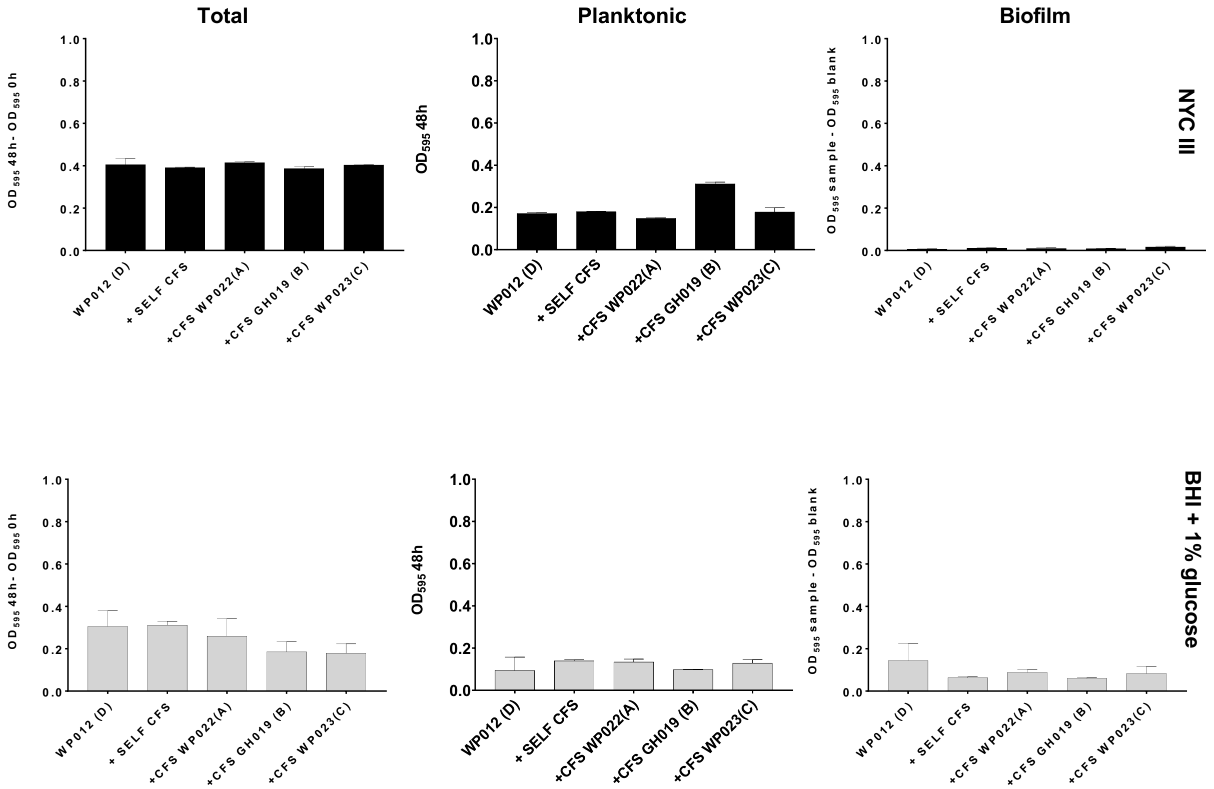

Fig S14: Growth of WP012 (subgroup D) in two different media: NYC III (upper panels, black bars) and BHI with 1% glucose (lower panels, grey bars). NYC III and BHI were spiked with 10% self-CFS, CFS of WP022 (subgroup A), GH019 (subgroup B) and WP023 (subgroup C) to challenge WP012. Total growth, planktonic growth and biofilm formation were measured at 48 h as described in the text. Error bars represent standard deviations of triplicates.

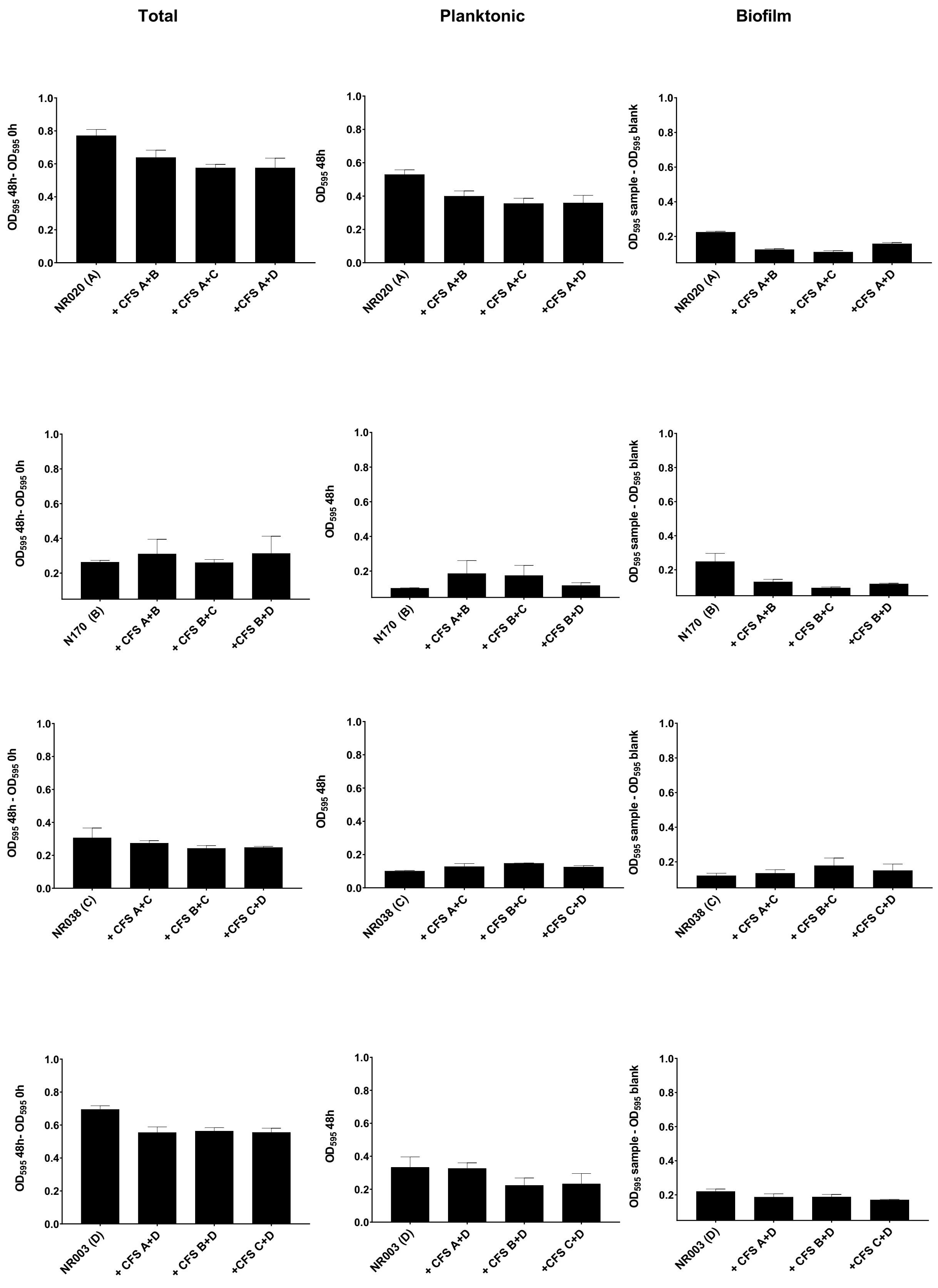
Total
Planktonic
Biofilm
OD_{595} 48h- OD_{595} 0h
OD_{595} 48h
OD_{595} sample - OD_{595} blank
NR020 (A)
+ CFS A+B
+ CFS A+C
+CFS A+D
N170 (B)
+ CFS A+B
+ CFS B+C
+CFS B+D
NR038 (C)
+ CFS A+C
+ CFS B+C
+CFS C+D
NR003 (D)
+ CFS A+D
+ CFS B+D
+CFS C+D

Fig S15: Growth of four representative strains (NR020, N170, NR038, NR003) of all subgroups (A, B, C, D) grown in media (BHI+ 0.25% maltose) spiked with 10% *Gardnerella* co-cultures (A+B, A+C, A+D, B+C, B+D, C+D) CFS. Optical density (Abs 595nm) was measured. Error bars represent standard deviations of triplicates.
