# Supplementary material for: Competition Among Gardnerella Subgroups From the Human Vaginal Microbiome
Source: Front Cell Infect Microbiol. 2019 Oct 31;9:374. doi: 10.3389/fcimb.2019.00374 (PMC6834547; doi:10.3389/fcimb.2019.00374)
Supplement: Supplementary file 2 [file Table_1.pdf]

Supplementary Materials

Table S1: cpn60 subgroup affiliations and origin of selected isolates of *Gardnerella*

Isolate[1]	cpn60 Subgroup	Species[2]
GH005	A	*G. leopoldii*
N72		*G. swidsinskii*
WP021		*G. swidsinskii*
WP022		*G. swidsinskii*
**VN003**		***G. leopoldii***
NR015		Unknown
NR016		*G. swidsinskii*
NR017		*G. leopoldii*
NR019		*G. leopoldii*
**NR020**		***G. swidsinskii***
NR021		*G. swidsinskii*
GH019	B	*G. piotii*
N95		Genome species 3
**N170**		**Genome species 3**
GH020		*G. piotii*
NR026		Genome species 3
GH007		*G. piotii*
N144		Genome species 3
**VN002**		***G. piotii***
N165	C	*G. vaginalis*
**NR001**		***G. vaginalis***
GH021		*G. vaginalis*
ATCC14018		*G. vaginalis*
NR037		*G. vaginalis*
**NR038**		***G. vaginalis***
NR039		*G. vaginalis*
WP023		*G. vaginalis*
NR037		*G. vaginalis*
N160	D	Genome species 10
**WP012**		**Genome species 9**
**NR003**		**Genome species 8**
NR047		Unknown

[1]Isolates used in co-culture experiments are in bold.

[2]Species identification based on nearest neighbour in the cpnDB_nr database (version 20190305) downloaded from www.cpndb.ca
