# Supplementary material for: Competition Among Gardnerella Subgroups From the Human Vaginal Microbiome
Source: Front Cell Infect Microbiol. 2019 Oct 31;9:374. doi: 10.3389/fcimb.2019.00374 (PMC6834547; doi:10.3389/fcimb.2019.00374)
Supplement: Supplementary file 3 [file Table_2.pdf]

Table S2: Subgroup specific primers and probes from Balashov et al. (2014)

Subgroup	Clade	Primer/probe	Sequence (5`- 3`)	Length(bp)
C	1	Gv1_fuc1_S (Forward)	CCA GTC ATA AGT TTG CGT TTT ACC	24
C	1	Gv1_fuc1_AS (Reverse)	TGG CAC TGG CAA AGT TTA CAA C	22
C	1	Gv1_fuc1_TM	HEX-CTC GCC GCA AGC ACC ATC AAG CCA-BHQ1	24
B	2	Gv2_hyp_S (Forward)	GCA AAG CAG ACT GAG CGT ATT AG	23
B	2	Gv2_hyp_AS (Reverse)	GTA ATA ATC AGG CTC CTC ATC GC	23
B	2	Gv2_hyp_TM	HEX-CGC AGG CGC TCG CAT AAC AGT GCA-BHQ1	24
D	3	Gv3_thi_S (Forward)	TTC TGC TTC TTC TGC TAT TTG CTG	24
D	3	Gv3_thi_AS (Reverse)	TTC GTT GAC TTT TGG GCA ACA TG	23
D	3	Gv3_thi_TM	6-FAM-CGG TCC GTG CCG TTC ATT TGG TCC-BHQ1	24
A	4	Gv4_cic_S (Forward)	CCT ACG CAA GCT CCA GAC GAC	21
A	4	Gv4_cic_AS (Reverse)	ACA AGT TGC ACT CTT CGA GCT GG	23
A	4	Gv4_cic_TM	6-FAM-ACT CGG CTG AAG CAC ACC ACC ACT-BHQ1	24
